# Gene-based Higher Criticism methods for large-scale exonic single-nucleotide polymorphism data

**DOI:** 10.1186/1753-6561-5-S9-S65

**Published:** 2011-11-29

**Authors:** Shiquan He, Zheyang Wu

**Affiliations:** 1 Department of Mathematical Sciences, Worcester Polytechnic Institute, 100 Institute Road, Worcester, MA 01609-2280, USA

## Abstract

In genome-wide association studies, gene-based methods measure potential joint genetic effects of loci within genes and are promising for detecting causative genetic variations. Following recent theoretical research in statistical multiple-hypothesis testing, we propose to adapt the Higher Criticism procedures to develop novel gene-based methods that use the information of linkage disequilibrium for detecting weak and sparse genetic signals. With the large-scale exonic single-nucleotide polymorphism data from Genetic Analysis Workshop 17, we show that the new Higher-Criticism-type gene-based methods have higher statistical power to detect causative genes than the minimal *P*-value method, ridge regression, and the prototypes of Higher Criticism do.

## Background

Genetic factors within genes, pathways, or other physical and functional genome segments often jointly affect the pathogenesis of a disease. Compared with single-locus association analysis in genome-wide association studies, gene-based multiple-loci analysis explores the joint effect of genetic factors related to these functional units and thus likely better reveals the biomedical mechanism of complex traits. Because gene-based methods are potentially more powerful in discovering the complex genetic signals of common diseases, they are considered promising for the study of genetic associations [1]. In recent literature, a number of gene- and pathway-based methods have been studied [2-10]. However, they either assume independent loci [6,9] or have no theoretical justification for ways of considering linkage disequilibrium (LD) [3]. Because of the physical or genetic closeness of the grouped loci, LD is a significant and informative characteristic of genomic segments of genes. LD is particularly significant among genetic factors obtained from next-generation sequence data, for example, rare single-nucleotide polymorphism (SNP) variations located within the exome, which can hardly be replaced by tagging SNPs without losing information. Thus, to better reveal causative genes of complex diseases, investigators strive to properly use the information of LD by developing methods based on a solid statistical theory of multiple-hypothesis testing.

In the latest development of multiple-hypothesis testing theory, the Higher Criticism (HC) and innovated Higher Criticism (iHC) methods have proved to be asymptotically the most efficient hypothesis-testing procedures [11,12]. These methods sustain the lowest boundary for the weakness and sparsity of signals, below which signals are statistically undetectable. This property is important for genome-wide association studies because the unfound genetic factors are likely to be sparse (i.e., true genetic factors are only a small proportion of all considered factors) and weak (i.e., statistical associations of true genetic factors are weak at the population level because of small genetic effects or rare variations). Furthermore, HC-type procedures can incorporate the correlations into test statistics to provide higher statistical power in many circumstances. Based on this celebrated advance of multiple-testing theory, we propose to develop more powerful HC-type gene-based methods that incorporate LD information within genes. Using the large-scale exonic SNP data from Genetic Analysis Workshop 17 (GAW17), we compare the prototypes and extensions of HC-type methods with the single-SNP method and ridge regression. The results show that the original HC and iHC methods are not superior to the single-SNP method and ridge regression, but two new methods, eigen-decomposition Higher Criticism (eHC) and innovated Higher Criticism based on marginal estimation of genetic effects (iHCM), show better performance.

## Methods

We applied six gene-based methods to analyze the GAW17 data of 697 unrelated individuals from the pilot3 study of the 1000 Genomes Project. Each method was applied to each of the 200 replicates of simulated Q1, Q2, and binary traits. Their ability to detect causative genes was compared on the basis of the average performance over the 200 replicates. Genotype was coded as the number of major alleles at each SNP.

### Method 1: minimal *P*-value method

The minimal *P*-value method is a gene detection method that uses single-SNP analysis. The association of individual SNPs in a gene are tested, and the smallest *P*-value (or, equivalently, the test statistic with the largest magnitude) is used to study the significance of that gene. For quantitative traits, the *T*-test statistic from a simple regression model is used. For the binary outcome, a *Z* statistic is used [13].

### Method 2: ridge regression

Ridge regression is a natural and powerful approach for testing the association between a response and a group of correlated covariates. Here, we apply ridge regression to detect gene-based association, accommodating LD among SNPs within genes. To our best knowledge, we did not find in the literature any application of ridge regression to testing for association of grouped SNPs. Our simulations (not reported here) show that in many situations ridge regression has a higher statistical power than many LD-incorporating methods proposed in the literature, such as principal components analysis [2,7], linear and quadratic combination tests, and the decorrelation test [3]. To estimate the vector of ridge regression coefficients, we obtain the tuning parameter by minimizing the cross-validation prediction (R function lm.ridge [14]). The sum of the squared residuals describing the goodness-of-fit of the model is treated as the score for each gene. For simplicity, the same procedure is applied to both quantitative and binary traits.

### Method 3: Higher Criticism

The Higher Criticism is a multiple comparisons procedure initialized by Tukey [15] and further developed by Donoho and Jin [11]. We adapt this procedure as a gene-based method to detect the significance of the association between a gene and a trait. For genes containing multiple SNPs, the rejection of the association between a gene and a trait is equivalent to the rejection of the joint null hypothesis between multiple SNPs in the gene and the trait. Let *p*_(1)_ ≤ *p*_(2)_ ≤ … ≤ *p*_(_*_L_*_)_ be the ordered *P*-values of *L* individual SNPs in a gene, as described in method 1. The HC statistic for this gene is:(1)

The idea of HC is to find the largest standardized difference between the observed and expected fractions of significance under the joint null hypothesis over a range of significance levels. It provides proper family-wise error control for simultaneously testing multiple hypotheses. Under the assumption of independence among tests, Donoho and Jin [11] proved that HC is optimal in the sense that among all testing procedures, HC provides the lowest boundary for the region of amplitude versus sparsity in which the procedure has asymptotically full statistical power. In the GAW17 data, because most genes have a small number of SNPs, we replace the lower bound of the significance range 1/*L* with 10^−10^.

### Method 4: innovated Higher Criticism

To exploit the potential advantages that correlations among test statistics can offer, Hall and Jin [12] developed innovated Higher Criticism (iHC) to incorporate the information of correlations. They showed that iHC can provide higher power when the correlation matrix is a Toeplitz matrix or when the correlation decays at a polynomial rate along with the distance between the correlated tests. To adapt iHC into the gene-based detection, we use the following procedure:

**Step 1.** Jointly estimate the vector of regression coefficients for the SNPs in a gene, such that . For quantitative traits, the least-squares estimation is applied so that:(2)

where *X* is the design matrix of genotypes. Because *σ*^2^ is unique for a specific outcome variable, it will not affect the comparison of the statistics and can thus be treated as having a value of 1. For the binary trait, we fit a multicovariate logistic regression to get the maximum-likelihood estimator of the coefficient vector and its variance matrix.

**Step 2.** Let:(3)

be an inverse matrix (lower triangular) of the Cholesky factorization of , that is, . We trim *U* by making the lower off-diagonals zero if they are equal to or more than *b* rows away from the central diagonal. Specifically, the resulting matrix is:(4)

where for some bandwidth 1 ≤ *b* ≤ *n*,  if *k* − *b* + 1 ≤ *j* ≤ *k*, and  otherwise. Hall and Jin [12] suggested using use *b* = log *n*. Here we choose *b* as the largest integer equal to or less than log *n*.

**Step 3.** We get matrix  by normalizing each column of  such that the L2 norm of each column of  is 1. Let .

**Step 4.** The iHC statistic is the HC statistic based on:(5)

### Method 5: eigen-decomposition Higher Criticism

The key idea of iHC is to transform the correlated tests into independent tests so that the HC procedure is valid to apply. The transformation process incorporates the correlation information into the new test statistics. Specifically, Cholesky decomposition allows  to be approximately *N*(*Vβ*, *I*), where *I* is an identity matrix. Note that when the trimming bandwidth *b* = 1, we have *V* = *U*. Here we consider eigen-decomposition, which can also provide the transformation into independence. The steps are as follows.

**Step 1.** Estimate the coefficients  and the corresponding covariance  of the SNPs in a gene as in step 1 of method 4.

**Step 2.** We take eigen-decomposition:(6)(7)

where *Q* is a matrix whose columns are eigenvectors of  and Λ is a diagonal matrix whose diagonal elements are the eigenvalues corresponding to the eigenvectors. Note that we have:(8)

which satisfies the assumption of independent normality in the theoretical research of Donoho and Jin [11].

**Step 3.** The eHC statistic is the HC statistic based on the *P*-values:(9)

### Method 6: innovated Higher Criticism based on marginal estimation

Because many SNPs in the GAW17 data have rare minor alleles, the joint estimation of genetic effects by multiple covariate (logistic) regression is unstable (imagine a high-dimensional contingency table of many cells being 0). To accommodate this issue, we studied innovated Higher Criticism based on marginal estimation of genetic effects (iHCM). Specifically, we obtain the test statistics for SNPs in a gene using single-SNP analysis (as in method 1, *T*-test statistics are used for quantitative traits; *Z* statistics are used for the binary disease liability).

For both quantitative and binary traits, we consider two approaches to estimate the correlations among the test statistics. In the first approach, from each data replicate we calculate one set of marginal *T* or *Z* statistics and then combine the 200 sets of test statistics from the 200 replicates to calculate the Pearson correlation coefficients. In the second approach, we approximate the correlation between two test statistics by using the correlation between the genotypes of these two SNPs. The second method is not quite proper, especially under nonnull situations, but it has been widely used in the literature [6,16]. Based on our experience, the first method is slightly better. The results reported later in this paper are based on the first estimation approach. Using  to denote the marginal *T*- or *Z*-test statistics and  to denote the estimated covariance matrix of  (because the marginal test statistics are asymptotically standard normal distributed under the null hypothesis), we applied steps 2–4 from method 4 to obtain the iHCM statistic.

### Approaches for comparison among genes

We consider two permutation-based approaches to compare the significance of different genes adjusted for the distinct number of SNPs and their correlation structures. In each permutation, we randomly shuffle the response and then calculate all the test statistics from methods 1–6 for all genes. Let *S_i_* and , *i* = 1, …, *I*, *j* = 1, …, *J*, denote any test statistic from methods 1–6 for the *i*th gene from the original data and from the *j*th permutation. The first approach calculates the empirical *P*-values for each gene:(10)

The second approach normalizes the scores from Eq. (10) to make them directly comparable among genes. Specifically, the normalized scores are:(11)

where  is a vector of the test statistics of gene *i* from permutations. For both approaches, the number of permutations is *J* = 10,000. To simplify the computation, the statistics obtained from permuting replicate 1 are also used to get the empirical *P*-values and the normalized scores for other replicates, because the null distributions for all replicates are the same.

Note that our knowledge of the underlying causative genes was used only for the purpose of evaluating the performance of the gene detection methods. The design of the analysis methods does not rely on knowing which genes are true.

## Results

The genotype data were cleaned in the following way before analysis. If the vectors of SNP genotypes in a gene were linearly dependent, we removed SNPs with the smallest minor allele frequencies (MAFs) until the remaining SNP genotype vectors became linearly independent. This process avoided overfitting the models and retained the full genetic information of each gene because the deletion did not change the linear space expanded by the SNP genotype data. We analyzed 23,980 SNPs after 507 redundant SNPs (not including any causative SNPs) were removed. Most of the remaining SNPs had very small MAF: Both the minimum and the 1st quantile were 0.000717, the median was 0.002152, the mean was 0.030630, the 3rd quantile was 0.01076, and the maximum was 0.4993.

**Figure 1:**
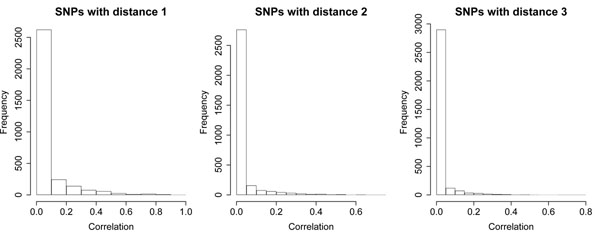
**Distribution of correlation magnitude between SNPs**. We count the correlation between a pair of SNPs if they are located in the same gene and have a distance 1 (adjacent), 2 (separated by one SNP), or 3 (separated by two SNPs).

The number of SNPs and the LD structure within genes are two critical factors for gene-based methods. The GAW17 data contain 3,205 genes. For the distribution of SNP numbers within genes, both the minimum and the 1st quantile were 1, the median was 2, the mean was 7.5, the 3rd quantile was 8, and the maximum was 205. The distribution of the absolute Pearson correlation coefficients between all pairwise SNPs within genes also skewed to the right: The median was 0.008752, the mean was 0.05682, the 3rd quantile was 0.04558, and the maximum was 0.9606. Figure 1 shows the distributions of the correlations between SNP pairs that are located within the same genes and have distances 1 (adjacent), 2 (separated by one SNP), or 3 (separated by two SNPs). As shown in Figure 1, there is an overall trend of correlation decay when the distance between the SNPs increases. To some extent, this observation justifies the application of the iHC procedure, which asymptotically performs well when the correlation decays at a polynomial rate.

We compared the six methods described earlier according to how well they ranked genes. A gene with a smaller empirical *P*-value (or a larger standardized score) was more statistically significant and thus had a higher rank (i.e., a smaller value of rank). We prefer methods that give higher ranks to causative genes. For the quantitative traits Q1, Q2, and the binary disease liability, the table in additional file 1 lists the average and standard deviation of ranks (over 200 replicates) of causative genes based on empirical *P*-values. Different methods may provide distinct advantages for different genes. The means and standard deviations for the average ranks over all causative genes are also given in the table in additional file 1 and provide an overall evaluation for different methods. The smallest average ranks are boldfaced to highlight the best performances. From the table in additional file 1 we can see that the newly developed HC-based methods eHC and iHCM have better performance. Particularly for Q1, Q2, and the binary trait, the iHCM method is uniformly the best. For Q2, the eHC method also performs well. The original HC method [11] and the iHC method [12] are not superior to the minimal *P*-value method and ridge regression. Our results for the comparison of the standardized scores to the ranked genes give similar patterns.

## Discussion

For the large-scale exonic SNP data of unrelated individuals and the 200 simulation replicates of quantitative and binary traits, most of the true genes are not ranked at the top. Taking the binary trait as an example, we find that the mean of the average ranks obtained from the minimal *P*-value method is even larger than a value that would be obtained by random choice (≈1,600, or half the total gene number). One of the potential causes is rare variations. In the GAW17 data most of the causative SNPs have very small MAFs (). SNPs with rare variations are difficult to detect for two reasons. First, the statistical signals are weak; and, second, the traditional association measurements, such as those based on regression or logistic regression, do not provide stable estimates, which is evidenced by the large standard deviations for the ranks of true genes over 200 replicates in Table 1.

For the weak signal problem, HC-type methods are ideal for detecting weak and sparse signals. However, the stability problem affects any method developed on the basis of unstable estimations. Some methods that combine rare variations to stabilize the estimations (e.g., weighted-sum methods [17] or collapsing methods [18]) can be applied to improve the HC-type methods.

There are several gaps between GAW17 data and the assumptions of the HC-type methods. First, the theoretical study for HC-type methods is based on asymptotics and assumes a large number of tests in each group. However, the GAW17 data have relatively small numbers of SNPs in genes. Second, the iHC method assumes that the correlation matrix is a Toeplitz matrix or has elements that decay at a polynomial rate. Figure 1 does show an overall trend of decay, but the real correlations in specific genes are much more complicated. We expect that the original iHC method could perform better for those data from genome-wide association studies that are closer to these assumptions when they have more SNPs contained in genes and more consistent correlation decay (e.g., among further separated SNPs).

## Conclusions

Gene-based methods that properly incorporate linkage disequilibrium information can improve the detection of causative genes. In Genetic Analysis Workshop 17, we studied the large-scale exonic SNP data of unrelated individuals and the 200 simulation replicates of quantitative traits (Q1, Q2) and the binary trait. Two novel HC-type gene-based methods, eHC and iHCM, have better performances than the minimal *P*-value method (a benchmark), ridge regression (a good method accommodating linkage disequilibrium), and the original HC methods (HC and iHC) in that eHC and iHCM lead to higher average ranks of all causative genes.

## Competing interests

The authors declare that there is no competing interest.

## Authors’ contributions

SH and ZW participated in the design of the study, performed the statistical analysis, drafted the manuscript, read and approved the final manuscript.

## Supplementary Material

Additional file 1 Average and standard deviation of ranks of true genes over 200 replicates for Q1, Q2, and the binary trait. Click here for file
