# Supplementary material for: Gene-based Higher Criticism methods for large-scale exonic single-nucleotide polymorphism data
Source: BMC Proc. 2011 Nov 29;5(Suppl 9):S65. doi: 10.1186/1753-6561-5-S9-S65 (PMC3287904; doi:10.1186/1753-6561-5-S9-S65)
Supplement: Additional file 1 — Average and standard deviation of ranks of true genes over 200 replicates for Q1, Q2, and the binary trait. [file 1753-6561-5-S9-S65-S1.pdf]

Tables

Table 1 - Average and standard deviation of ranks of true genes over 200 replicates for Q1, Q2, and the binary trait

The last two rows in each section give the mean and the standard deviation of the average ranks over the group of all true genes. The ranking is based on empirical *P*-values.

	Minimal *P*-value		Ridge regression		HC		iHC		eHC		iHCM	
Trait	Average	SD	Average	SD	Average	SD	Average	SD	Average	SD	Average	SD
Q1												
ARNT	265	405	596	522	888	948	780	834	787	288	261	399
ELAVL4	1,044	778	954	727	971	773	892	666	956	298	1,070	656
FLT1	21	12	15	9	139	200	255	296	586	288	54	26
FLT4	971	700	882	679	842	657	789	573	437	152	695	574
HIF1A	810	676	923	665	886	739	802	660	342	211	678	612
HIF3A	1,874	784	1,856	801	1,723	790	1,498	813	1,219	197	1,508	665
KDR	104	166	40	79	113	107	193	83	253	144	240	208
VEGFA	1,129	703	931	694	991	712	544	498	335	176	945	661
VEGFC	631	608	615	614	694	591	1,154	651	2,074	1,201	824	736
Mean	761	537	757	532	805	613	768	564	777	328	**697**	504
SD	586	280	551	288	482	279	410	242	583	333	**459**	243

Q2												
BCHE	825	841	733	767	670	789	723	707	635	180	375	447
GCKR	629	728	624	734	676	723	1,200	668	2,001	808	769	773
INSIG1	1,739	870	1,783	870	1,754	889	1,326	1,014	441	577	1,660	887
LPL	869	818	882	817	850	860	673	716	643	215	582	560
PDGFD	741	675	722	661	700	687	561	561	794	309	517	500
PLAT	1,520	912	1,543	876	1,451	914	1,372	828	1,064	220	743	630
RARB	1,254	824	1,172	822	1,196	824	996	754	401	248	1,067	720
SIRT1	750	892	789	792	636	800	713	740	500	176	666	570
SREBF1	1,181	889	906	804	932	841	1,003	762	754	198	397	430
VLDLR	1,438	953	1,333	898	1,261	948	1,237	843	686	265	728	654
VNN1	168	357	229	407	505	742	378	626	463	409	250	400
VNN3	432	558	324	498	395	553	295	392	631	286	237	300
VWF	1,221	886	1,450	914	1,209	949	1,060	858	1,271	345	1,473	823
Mean	982	785	961	758	941	809	887	729	791	326	**728**	592
SD	455	168	469	153	403	113	356	153	440	182	**439**	177
Binary trait												
ARNT	1,986	816	1,315	899	1,455	953	1,072	891	1,065	879	831	474
ELAVL4	2,082	503	913	721	928	665	1,161	988	1,209	957	686	411
FLT1	2,747	404	251	410	208	357	250	465	314	541	348	249
FLT4	1,880	740	1,623	867	1,749	803	2,048	614	1,999	647	1,815	654
HIF1A	1,708	580	1,908	811	1,887	832	2,052	629	2,077	631	1,649	594
HIF3A	1,766	835	1,393	849	1,309	883	1,671	957	1,666	961	1,121	472

KDR	2,126	489	1,062	851	1,079	812	1,775	551	1,749	568	1,314	677
VEGFA	1,809	740	1,778	831	1,670	868	1,865	790	1,865	796	1,916	668
VEGFC	2,451	719	1,330	153	1,339	155	1,425	980	1,437	980	2,554	595
BCHE	2,551	565	1,251	908	1,352	902	1,719	295	1,712	317	1,035	678
GCKR	1,368	874	1,847	933	1,789	895	1,765	1,026	1,759	1,032	2,013	801
INSIG1	2,444	423	963	724	1,110	826	1,375	891	1,379	907	864	525
LPL	1,844	773	1,322	822	1,358	831	1,355	874	1,337	881	668	406
PDGFD	1,346	741	1,878	849	1,913	871	1,942	978	1,919	964	1,772	693
PLAT	1,505	887	1,633	830	1,396	858	1,702	597	1,847	433	1,022	481
RARB	1,579	532	1,273	740	1,166	728	1,829	729	1,844	736	140	192
SIRT1	1,985	748	1,456	888	1,303	858	594	685	1,324	690	932	661
SREBF1	1,578	903	1,414	896	1,173	851	1,335	675	1,665	697	1,015	522
VLDLR	1,840	833	1,588	883	1,556	939	1,563	776	1,473	763	791	453
VNN1	1,839	890	1,378	645	1,370	926	1,450	935	1,209	969	1,245	656
VNN3	1,701	737	1,516	906	1,469	928	1,183	955	1,553	942	994	545
VWF	1,305	857	1,754	843	1,867	950	1,534	910	1,553	931	1,977	773
AKT3	1,748	895	1,567	900	2,388	405	1,526	148	1,677	148	1,997	774
BCL2L11	1,384	1,110	2,012	869	2,006	905	1,681	629	1,909	643	1,432	623
ELAVL4	2,082	503	913	835	928	665	1,854	988	1,209	957	686	411
HSP90AA1	2,332	643	1,250	721	1,064	931	1,161	818	1,593	820	735	441
NRAS	1,979	545	1,374	152	1,357	155	1,461	787	1,804	791	1,703	743
PIK3C2B	2,232	724	779	661	680	597	1,821	763	1,332	757	97	73
PIK3C3	1,949	595	1,247	795	1,204	814	1,321	914	1,282	925	1,121	613
PIK3R3	1,331	424	1,888	671	1,966	651	1,329	648	2,152	644	1,136	538

PRKCA	2,499	612	630	663	587	680	2,160	723	602	748	793	698
PRKCB1	1,588	881	1,971	794	1,680	954	1,441	968	1,505	952	849	418
PTK2	1,847	503	1,789	846	1,619	873	1,815	379	1,819	336	2,000	752
PTK2B	1,624	881	1,599	900	1,485	934	1,572	960	1,660	978	1,779	766
RRAS	1,743	928	1,556	935	1,622	926	1,596	956	1,575	959	1,892	807
SHC1	1,488	979	1,843	932	1,777	1,011	1,359	1,003	1,368	1,008	998	560
SOS2	2,308	742	761	786	963	829	664	769	693	773	315	149
Mean	1,880	718	1,406	776	1,399	784	1,498	774	1,517	775	**1,196**	555
SD	376	177	411	184	434	209	404	213	398	214	**596**	183
